# Predetermined Flake Production at the Lower/Middle Paleolithic Boundary: Yabrudian Scraper-Blank Technology

**DOI:** 10.1371/journal.pone.0106293

**Published:** 2014-09-05

**Authors:** Ron Shimelmitz, Steven L. Kuhn, Avraham Ronen, Mina Weinstein-Evron

**Affiliations:** 1 Zinman Institute of Archaeology, University of Haifa, Haifa, Israel; 2 School of Anthropology, University of Arizona, Tucson, Arizona, United States of America; University of Oxford, United Kingdom

## Abstract

While predetermined *débitage* technologies are recognized beginning with the middle Acheulian, the Middle Paleolithic is usually associated with a sharp increase in their use. A study of scraper-blank technology from three Yabrudian assemblages retrieved from the early part of the Acheulo-Yabrudian complex of Tabun Cave (ca. 415–320 kyr) demonstrates a calculated and preplanned production, even if it does not show the same complexity and elaboration as in the Levallois technology. These scraper dominated assemblages show an organization of production based on an intensive use of predetermination blank technology already in place at the end of the Lower Paleolithic of the Levant. These results provide a novel perspective on the differences and similarities between the Lower and Middle Paleolithic industries. We suggest that there was a change in the paradigm in the way hominins exploited stone tools: in many Middle Paleolithic assemblages the potential of the stone tools for hafting was a central feature, in the Lower Paleolithic ergonometric considerations of manual prehension were central to the design of blanks and tools.

## Introduction

The Yabrudian is one of three facies (or industries) that make up the Acheulo-Yabrudian cultural complex. This group of assemblages is generally dated between 415-250/220 kyr [1]–[4]. Its three facies, defined according to the frequencies of key artifact forms, are the *Acheulian* (also known as Acheulo-Yabrudian) with conspicuous handaxe manufacture and flake production, the *Yabrudian* with numerous scrapers made on large flakes, and the *Amudian* (sometimes called Pre-Aurignacian) with conspicuous blade production and numerous ‘Upper Paleolithic’ tool types [5]–[6]. In the past these facies were thought to represent different cultures [7]–[8] and even hominin types [9]–[10]. Today, following Jelinek [11]–[13] and Copeland [14]–[15], most researches assume that the three assemblage types represent different variants of a single cultural complex, perhaps reflecting fluctuating activity frequencies. The concept of the Acheulo-Yabrudian as a single heterogeneous culture complex is also supported by recent studies demonstrating spatial differentiation [16] in assemblage composition within a single layer, as well as continuity in technological knowledge and performance among the three facies [17].

The Acheulo-Yabrudian complex has been assigned to both the Middle Paleolithic (e.g. [18]–[19]) and the Lower Paleolithic [8], [20] by different researchers. Today most scholars would assign it to the late Lower Paleolithic ([5], [6], [21]; but see [22]). However, it is of little consequence whether one assigns the Acheulo-Yabrudian to either of these broad chrono-stratigraphic categories: the assemblages making up this complex share many similarities with both the Lower and Middle Paleolithic ([17], pp. 333–353; [23]) and are chronologically and stratigraphically intermediate between them.

The phenomenon of “predetermination”, standardization of blank morphology achieved by shaping the core, has received considerable attention from Paleolithic scholars in the past decades. Because it appears to embody a degree of forward thinking, predetermination is thought to have particular mental pre-requisites (e.g. [24], pp. 187–189). Predetermination is well-expressed in the Middle Paleolithic through the frequent but geographically variable use of the Levallois method (e.g. [25]–[27]). Use of predetermined debitage technology is even considered by some to be one of the main characteristics differentiating the lithic industries of the Middle Paleolithic from the previous period (e.g. [28]–[30]). Lahr and Foley [31], who refer to it as ‘Technological Mode 3’, argue that this change attests to the growing cognitive capabilities of the hominins who produced the assemblages. However, it is clear that technologies involving some degree of predetermined debitage emerged in the Lower Paleolithic, as exemplified by the Kombewa technique ([32], pp. 68–70), the various Acheulean large flake manufacture schemes (e.g. [33]–[34]) and even some very early examples of Levallois technology (e.g. [35]–[37]).

It has also been claimed that the Amudian, with its systematic blade production, indicates a shift in the organization of lithic technology toward predetermined production at the end of the Lower Paleolithic in the Levant [17],[38]. The Amudian however, is actually the rarest of the three facies that comprise the Acheulo-Yabrudian complex; most assemblages attributed to this complex are of the Yabrudian or the Acheulean facies [5]–[8], [39]–[46]. Moreover, while Amudian technology has been the topic of several studies (e.g. [17], [38], [47]–[49]), technological research of the other two facies of the Acheulo-Yabrudian complex is more limited [16], [50] (although data on bifaces from these facies can be found; e.g. [51]–[53]).

The present study aims to fill in some of the gaps in knowledge about these late Middle Pleistocene assemblages. Using three assemblages from the excavations at Tabun Cave by Arthur Jelinek [11] and Avraham Ronen [54] we examine the *chaîne opératoire* for manufacture of scraper blanks. Based on the presence of a significant number of *débordants* and centripetal cores of different types in almost every assemblage from the Acheulo-Yabrudian layers of Tabun Cave we hypothesized that Yabrudian scraper blank production was also characterized by a kind of predetermined debitage technology. Because most Yabrudian tool assemblages are dominated by large scrapers (e.g. [6], [15], [20], [50], [55]), this would mean that a technological organization based on regularly predetermined reduction appeared with the Acheulo-Yabrudian complex, by 400 kyr if not earlier. Predetermination is present to some extent in all lithic reduction (e.g. [56]). What we argue however is that there was a systematic method of creating the scraper blanks, a repeated set of procedures aimed at producing blanks with specific morphological features. The presence of a well-articulated reduction sequence further implies that certain principles and procedures existed in the minds of the knappers. The results of the study provide a novel perspective on the differences and similarities between the Lower and Middle Paleolithic industries.

### The Acheulo-Yabrudian complex of Tabun Cave and the samples analyzed

**Figure 1:**
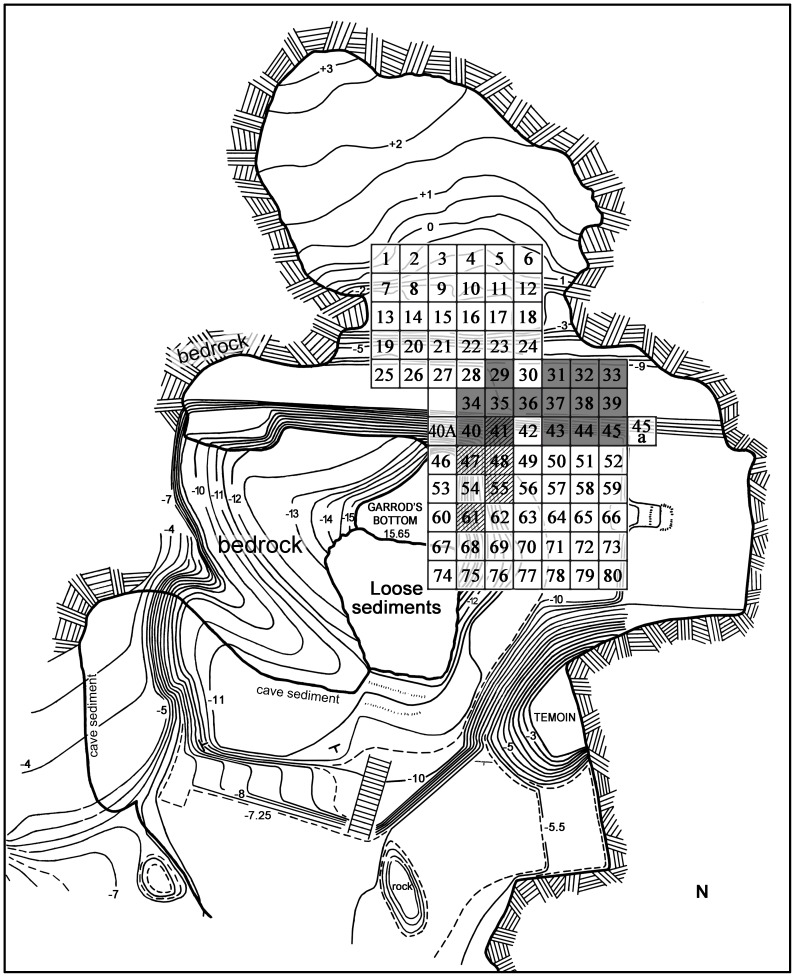
Map of Tabun Cave with excavated squares and location of the sampled layers: Beds J82BS and J83B1 in gray, R63 in dashed line. The cave outline is based on a map by Goldberg [101, pl. 1].

**Figure 2:**
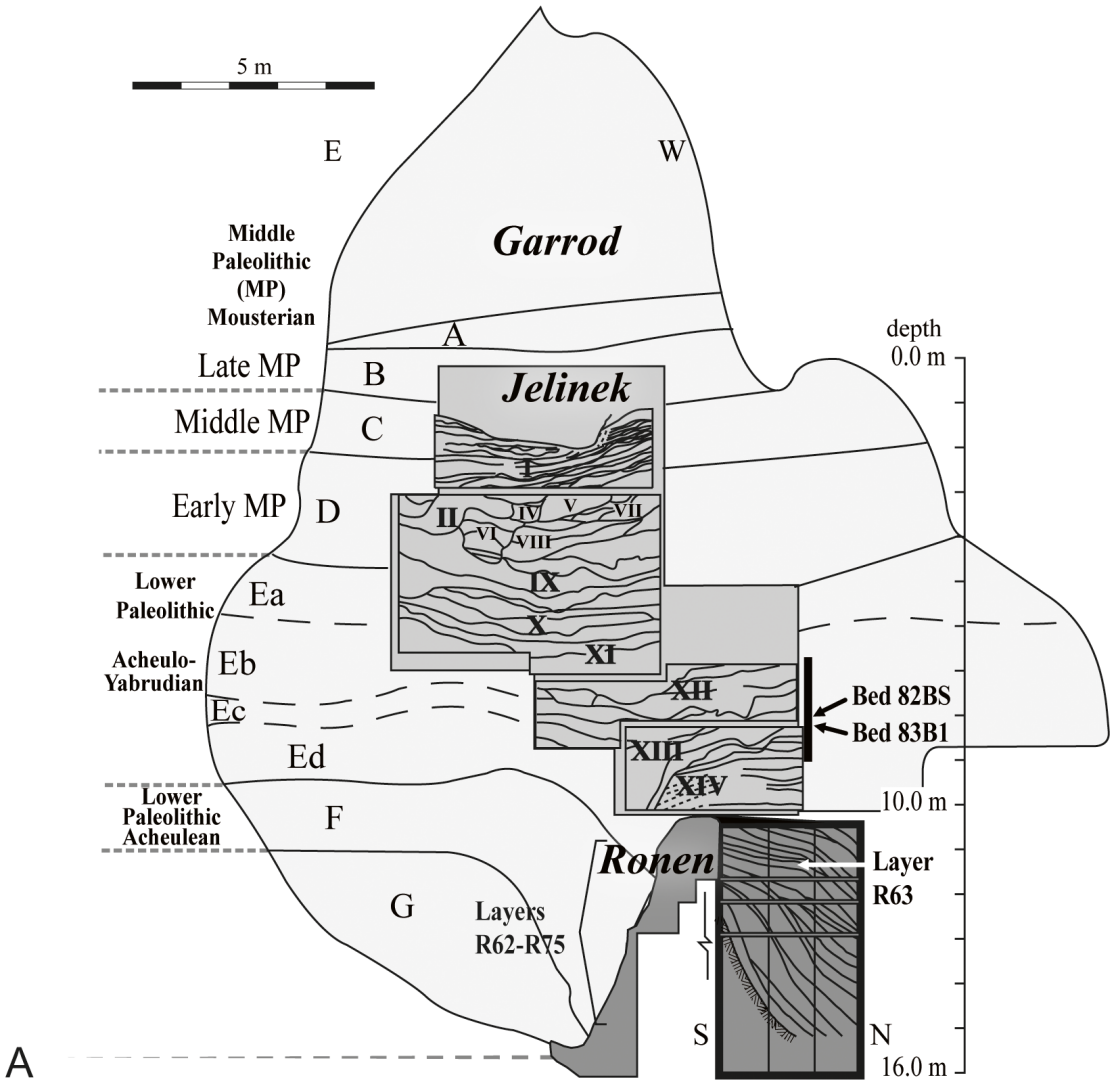
Tabun section showing the general location of the studied samples.

Tabun cave, Mount Carmel, Israel, contains a uniquely long sequence of archaeological layers extending ca. 25 m, beginning at the Lower Paleolithic and ending at the late Mousterian Middle Paleolithic. The results of Garrod's excavations at the site (1929–1934) led to the division of its stratigraphic sequence into seven thick, heterogeneous layers. The more recent excavations at the site by Jelinek (1967–1971) and Ronen (1975–2003) provide a more precise division of the stratigraphic and cultural sequences [11]–[13], [54]. In this paper we focus on the Acheulo-Yabrudian complex, which comes from Garrod's Layer E, Jelinek's Units X-XIV and Ronen's Layers R47-R63 (Figs. 1–2; layers/beds from Jelinek's and Ronen's excavations are denoted with the letters ‘J' and ‘R', respectively). Although recently some of the material from Ronen's excavations was ascribed a different terminology of layer division based on computer analysis of clustering of finds [54], [57]–[58] we prefer to employ the original division of layers based on sedimentological differences devised by Ronen during the excavation (e.g. [59]).

The high degree of variability within this part of the Tabun sequence was first noted by Garrod, who divided Layer E into four sub-layers [20]. She provided even more details regarding the fluctuations between scrapers, handaxes and blades [7] following Rust's [8] publication of Yabrud I, one of the small number of other deeply-stratified Acheulo-Yabrudian sites. Jelinek [6], [12]–[13] identified within his Unit X the Acheulean facies; in Unit XI the Acheulean, Yabrudian and Amudian; in Unit XII the Acheulean facies; and in Unit XIII the Yabrudian. Jelinek observed continuity in many aspects of typology and technology among the assemblages, leading him to unite the three of them under the term ‘Mugharan Tradition'. Assemblages from Unit XIV were originally considered to represent something distinct [13]. However, our current analysis of the lithics shows that they possess many features in common with the Acheualean and Yabriduan facies elsewhere in the sequence. A geoarchaeological study also described the sediments of Unit XIV as typical of the lower part of the Acheulo-Yabrudian layers [60].

The focus of this study is on three large Yabrudian collections, from Beds J82BS and J83B1 in Jelinek's excavation and Layer R63 in Ronen's excavation (Figs. 1–2). Beds J82BS and J83B1 consist of segments of layer J82-J83 of Unit XIII [12]–[13]. Bed J82BS extends over 10 m^2^ between elevations 7.27 and 8.40 m below datum. The layer inclines towards the north and ranges in thickness between 0.18 to 0.32 m. Four TL dates (on burnt flints) were obtained from Unit XIII with a mean of 302±27 kya which is unlikely since it is younger than the overlying Unit XII (324±31 kya) [1]. Three of these dates (T63, T64, T68) originated from the upper part of Unit XIII, Bed J81, which suffered some erosion before Unit XII formed; all gave relatively young dates: 280–290 kya. The fourth date (T67) from Bed J83 gave a date of 357±33 kya. A more precise date of J82 within this sequence cannot be determined since the two groups of dates (Units XII-XIII) are statistically indistinguishable. Bed J83B1 extends over 14 m^2^ of the excavation's grid, between elevations 7.62 m to 8.50 m below datum; it too inclines northward. Its maximal thickness is ca. 0.45 m, although it is mostly shallower (0.2–0.3 m). Layer J83 has provided a TL date of 357±33 kya [1].

Ronen's Layer R63 was excavated at the lowest step of the preserved stratigraphic section and covers 5 m^2^ of the excavation grid between elevations 10.07 m to 12.90 m. In the two eastern squares (sq. 41, 48) it is moderately inclined towards the north-west and the sinkhole in the central chamber. Its maximum thickness reaches ca. 0.9 m. Within squares 55 and 61 the inclination towards the sinkhole is more severe. The lower part of the section is not dated. A TL date of 415 kyr was obtained from Unit XIV [2]. While the correlation between the lower part of the section excavated by Ronen and Unit XIV is still not fully clarified due to the sharp, multi-directional inclinations of the layers, this part of Ronen's excavation is roughly contemporaneous with or slightly earlier than Jelinek's Unit XIV. Layer R63 is also placed lower than the layers excavated in square 87 near the eastern wall of the cave, which were dated by ESR/U-series (on teeth) to 387^+49^_-36_ kya [61]. The exact correlation between the two however is yet unclear. The layers below R63 show a gradual transition from the Acheulean *sensu stricto* to the Acheulo-Yabrudian [58], further supporting the idea that the age of these layers is greater than 415 kyr.

## Materials and Methods

The Israel Antiquity Authority site number of Tabun Cave is 1810/2 and its excavations were conducted under permits numbers C-108/1967, C-108/1968, C-108/1969, G-10/1970, G-8/1971 by Arthur Jelinek, and G-1/1975, G-39/1977, G-46/1978, G-35/1979, G-2/1980-01, G-5/1981, G-27/1982, G-52/1986, G-9/1988, G-61/1988, G-60/1991, G-54/1991, G-51/1992, G-95/1993, G-95/1994, G-109/1995, G-74/1996, G-99/1997, G-102/2000, G-98/2001, G-80/2002 by Avraham Ronen. The items retrieved from both excavations were recorded by serial numbers according to the square from which they were collected (Beds 82BS and 83B1: Sq 29, 31–41, 43–45; R63: Sq. 41, 47–48, 51, 55; see Fig. 1). The material from Jelinek's excavation is currently divided between the University of Arizona and the Israel Antiquities Authority. The presented study includes the part of the collection curated at the Department of Anthropology, University of Arizona (1009 E. South Campus Drive, Tucson, AZ 85721), as well as the material currently housed at the University of Haifa where the rest of the material is on loan from the Israel Antiquities Authority (Beth Shemesh Storage and Research Facility, Beth Shemesh Industrial Zone West; P. O. Box 586, 91004 Jerusalem). The material in the University of Arizona is catalogued according to beds and the analyzed material is registered as Bed 82BS and Bed 83B1. The material at the Israel Antiquities Authority is currently being organized into beds and layers as well; the analyzed material is already catalogued separately as Bed 82BS, Bed 83B1 and Layer R63.

Working on three large assemblages not only provides a perspective of the variability within the Yabrudian within the lower part of the sequence of Tabun, but also enables us to examine the correlation between the different patterns for a more accurate reconstruction of the reduction sequences. Since our goal is to reconstruct the organization of production, we recorded a similar range of attributes for both modified and unmodified blanks. This leads to a more complete reconstruction of the reduction sequence and at the same time provides a clear view of the criteria for selecting blanks for retouch or to be used as cores. A certain number of items were worked both as tools and as cores on flakes, some at different stages of their life histories: these were placed in the tool category and are further described in the text.

Below we first present the basic technological attributes of the three assemblages, including a short description of the retouched tools, which will serve as a base for reconstructing the reduction sequence. While Yabrudian assemblages with heavily-retouched scrapers are a fertile ground for studies of artifact life histories (e.g. [62]–[64]), our analysis here focuses on blank technology. Thus, in recording metrics we distinguish between measurements that are unaffected by or only slightly modified by retouch, and which are therefore representative of the original blanks, and measurements which have been significantly altered by retouch. In metrical analysis referring to blank size we are specifically concerned with the former. Length is measured perpendicular to the striking platform ([65], p.100, fig 5.8:C), width is measured parallel to the striking platform at the widest point. Thickness also records a maximum measurement.

Finds smaller than 2.5 cm were originally separated from the material of Jelinek's excavation and were not examined by us. The affect of this on the analysis however is minor since such items are not calculated among the general division of debitage and tools. In the case of the material from Ronen's excavation, only specific items smaller than 2.5 cm, including bladelets and spalls, were included in the general assemblages. General data of the small finds from Jelinek's excavation is provided with the courtesy of Michael Bisson. The sample of small pieces from bed J82BS includes 23 flakes, 3 thinning flakes, 4 blades, 21 trimming elements, 62 broken flakes, 10 broken blades, 2 core fragments, 35 angular pieces, 3 scraper fragments, 1 bifacial fragment, and 2 notch fragments. Bed J83BI includes 14 flakes, 8 thinning flakes, 4 trimming elements, 3 blade, 26 broken flakes, 1 broken blade, 2 core fragments, 20 angular fragments, 1 biface fragment, 1 scraper fragment and 1 notch fragment. None of these small items was added to the analysis since we could not assign them into the sub-categories within Table 1 which refer to blank type, state of preservation and secondary modification.

**Table 1:** The three Yabrudian assemblages.

		J82BS	J82BS	J82BS	J82BS	J82BS	J82BS	J83B1	J83B1	J83B1	J83B1	J83B1	J83B1	R63	R63	R63	R63	R63	R63
		whole	proximal	non-proximal	sum	%	%	whole	proximal	non-proximal	sum	%	%	whole	proximal	non-proximal	sum	%	%
PE flake	blank	21	4	3	28	30.1		56	5	4	65	39.6		44	10	12	66	51.2	
	c-o-f	2		1	3	3.2		2	1		3	1.8		8		2	10	7.8	
	shaped	53	2	7	62	66.7		78	7	11	96	58.5		46	1	6	53	41.1	
	sum	76	6	11	93	100	20.9	136	13	15	164	100	19.0	98	11	20	129	100	14.9
PE blade	blank	2		1	3	38		7	1	1	9	90		3	1		4	100	
	c-o-f				0	0.0					0	0.0					0	0.0	
	shaped	4		1	5	62.5		1			1	10.0					0	0.0	
	sum	6	0	2	8	100	1.8	8	1	1	10	100	1.2	3	1		4	100	0.5
flake	blank	45	7	3	55	34.8		141	27	24	192	53.0		159	37	54	250	62.2	
	c-o-f	3	1		4	2.5		5	1	2	8	2.2		17		5	22	5.5	
	shaped	86	3	10	99	62.7		123	11	28	162	44.8		99	9	22	130	32.3	
	sum	134	11	13	158	100	35.6	269	39	54	362	100	41.8	275	46	81	402	100	46.5
thinning flake	blank	1			1	50		38		2	40	95.2		15	2	2	19	86.4	
	c-o-f				0	0.0					0	0.0					0	0.0	
	shaped	1			1	50.0		1		1	2	4.8		3			3	13.6	
	sum	2	0	0	2	100	0.5	39	0	3	42	100	4.9	18	2	2	22	100	2.5
NBK-flake	blank	7	2		9	19.1		20	1	2	23	45.1		19	7	2	28	45.2	
	c-o-f	2		1	3	6.4		1			1	2.0		5			5	8.1	
	shaped	33	2		35	74.5		26		1	27	52.9		28		1	29	46.8	
	sum	42	4	1	47	100	10.6	47	1	3	51	100	5.9	52	7	3	62	100	7.2
NBK-laminar	blank	1			1	50		6			6	85.7		2	1	1	4	66.7	
	c-o-f				0	0.0					0	0.0					0	0.0	
	shaped	1			1	50.0		1			1	14.3		2			2	33.3	
	sum	2	0	0	2	100	0.5	7	0	0	7	100	0.8	4	1	1	6	100	0.7
blade	blank	1		1	2	40.0		14			14	56.0		21	1	4	26	81.3	
	c-o-f					0.0						0.0					0	0.0	
	shaped	3			3	60.0		9	1	1	11	44.0		6			6	18.8	
	sum	4	0	1	5	100	1.1	23	1	1	25	100	2.9	27	1	4	32	100	3.7
bladelet	blank													3	2	3	8	100	
	c-o-f																		
	tool																		
	sum													3	2	3	8	100	0.9
CTE	blank	21	2	1	24	42.1		40	2	5	47	51.1		44	1	7	52	65.8	
	c-o-f	1			1	1.8		6			6	6.5		4			4	5.1	
	shaped	31	0	1	32	56.1		31	2	6	39	42.4		21	1	1	23	29.1	
	sum	53	2	2	57	100	12.8	77	4	11	92	100	10.6	69	2	8	79	100	9.1
spalls	blank	8	1	0	9	90		10	0	1	11	91.7		38	2	4	44	100	
	c-o-f				0	0.0					0	0.0					0	0	
	shaped	1		0	1	10.0				1	1	8.3					0	0	
	sum	9	1	0	10	100	2.3	10	0	2	12	100	1.4	38	2	4	44	100	5.1
																			
sum	blank	107	16	9	132	34.6		332	36	39	407	53.2		348	64	89	501	63.6	
	c-o-f	8	1	2	11	2.9		14	2	2	18	2.4		34	0	7	41	5.2	
	shaped	213	7	19	239	62.6		270	21	49	340	44.4		205	11	30	246	31.2	
	sum	328	24	30	382	100	86.0	616	59	90	765	100	88.4	587	75	126	788	100	91.1
																			
core					51		11.5				68		7.9				64		7.4
core-tool					11		2.5				31		3.6				10		1.2
raw material							0.0				1		0.1				3		0.3
total					444		100				865		100				865		100
chunk					25						111						95		
chip																	374		
grand total					469						976						1334		

## Results

### General Features of the Yabrudian Assemblages

The basic composition of the three Yabrudian assemblages is presented in Table 1. Five round pebbles (J82BS: 2; J83B1: 2; R63: 1) that could have been used as hammer stones were collected but are not documented in the table.

Several raw material types are present in the assemblages, most of which could have been collected within a few km from the cave [66]. Raw material with calcareous, un-rolled cortical surfaces was preferentially selected (Table 2), indicating an effort to collect flint in fresh, pristine condition, a fact previously suggested by the study of the cosmogenic ^10^Be [67].

**Table 2:** Frequencies of type of cortex (including all items aside cores and core-tools).

	calcareous	patinated	rolled surface	n =
J82BS	89.8	3.8	6.4	266
J83B1	79.6	12.7	7.7	324
R63	83.5	12.6	3.9	357

**Figure 3:**
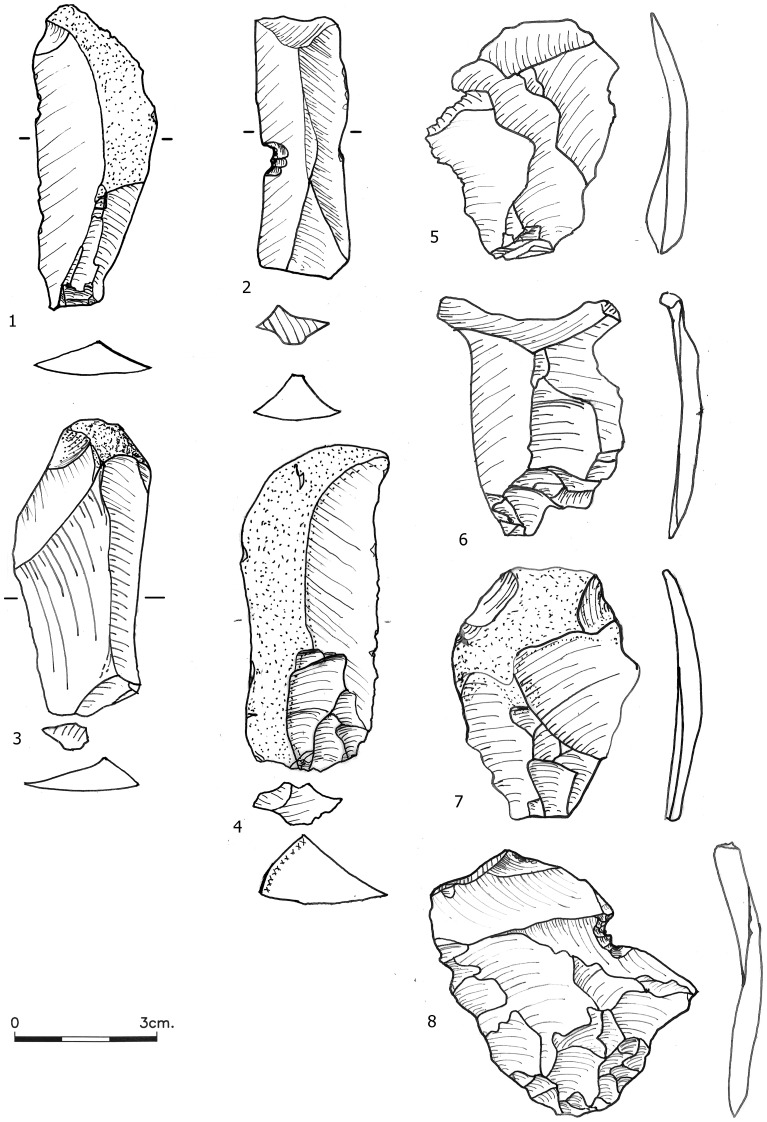
Blades (1–4) and thinning flakes (5–8) from Bed J83B1.

**Figure 4:**
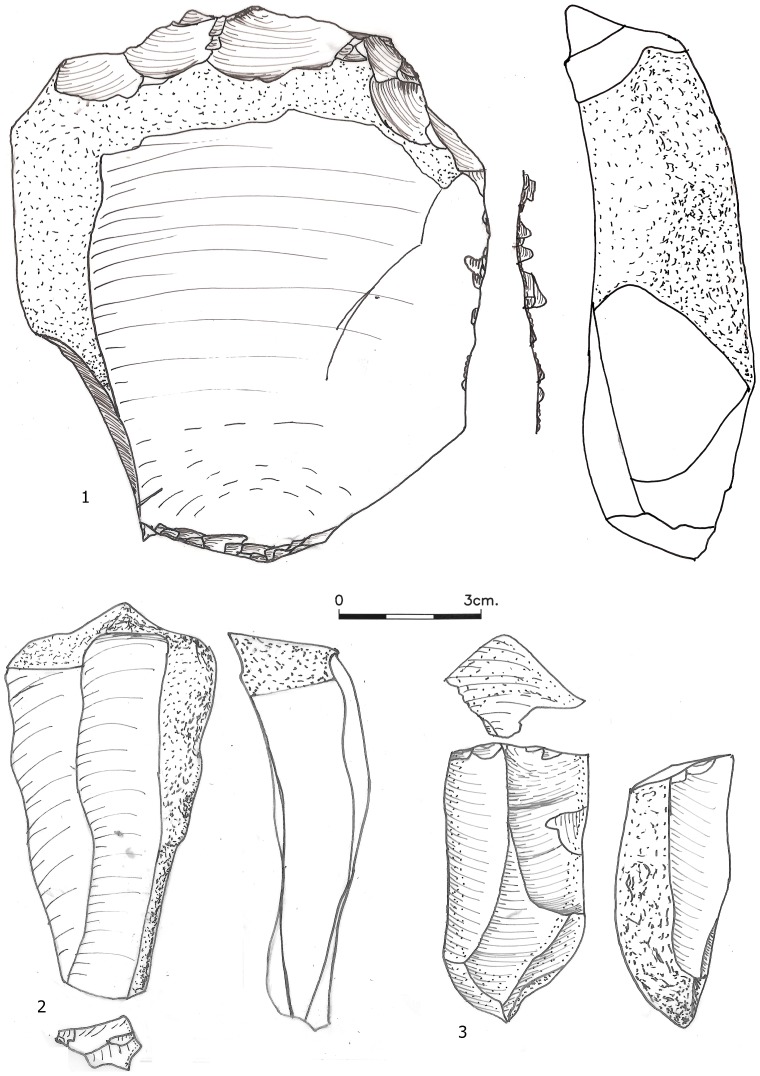
NBK-flake shaped into a denticulate (1), overpass item (2) and prismatic blade core (3) from Bed J82BS.

The three assemblages are composed of the waste and products of several reduction sequences. These include production of large flakes to serve as scraper blanks, simple flake production, blade production (Figs. 3–4), handaxe production and 'cores on flakes' (c-o-f) yielding very small products. While it is impossible to ascribe each of the items within these assemblages to a specific reduction sequence, it is still possible to draw several conclusions based on the character and frequencies of the most diagnostic blank types. Not surprisingly, these assemblages are dominated by flakes and flake tools reflected in the low index of laminarity (J82BS: 6.4; J83B1: 8.0; R63: 8.7; with reference to whole items only) and the small number of bifacial tools. The extremely low ratio of thinning flakes (Fig. 3:5, 6, 7, 8) to bifacial pieces (excluding small fragments of bifacials; J82BS: 0.3; J83B1: 1.6; R63: 2.4) indicates that the production of bifacials mostly occurred somewhere else. The few additional small thinning flakes found in the small finds would not change this ratio significantly. While the excavations within the cemented sediments often lead to higher breakage that could impede the identification of such delicate items, we recorded fragments as well as whole pieces so we suspect that the paucity of thinning flakes represents a genuine pattern.

**Figure 5:**
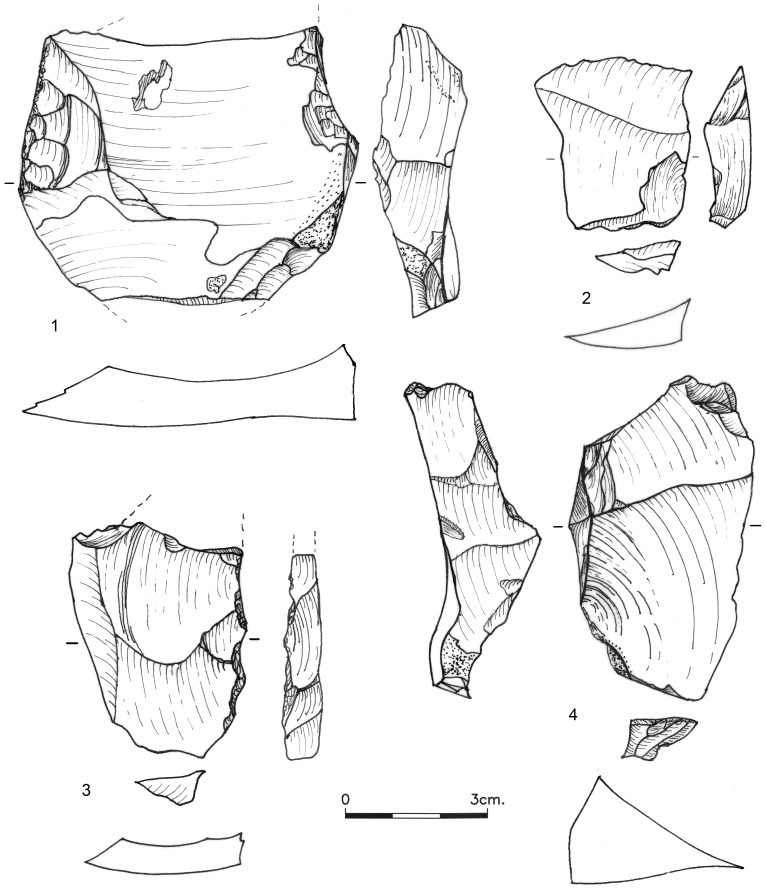
*Debordants*, Bed J82BS. No. 1 shaped into a scraper.

Primary element (PE) flakes (with cortex covering ≥30% of the dorsal face) and plain flakes are the prominent blank types in the assemblage, whether one is considering unmodified blanks or shaped items. Smaller amounts of cortex (<30% of the dorsal face) are also present on many of the flakes. Cortex on flakes is more common in Bed J82BS (44.1%) than in Bed J83B1 (32.6%) and Layer R63 (26.9%) (χ^2^ = 13.512, df = 1, p = 0.002; χ^2^ = 13.345, df = 1, p<0.0001, respectively). Naturally backed knives (NBKs) which have a strip of cortex along one edge with an angle ≥60° to the ventral face, are also common in all assemblages.

Striking platforms in all three assemblages usually consist of a single facet or old flake surface (Table 3). However, approximately one third of the striking platforms are dihedral, faceted or multi-scarred, evidence of more elaborated shaping. In this study, multi-scarred platforms differ from faceted ones in that they lack negatives of the bulbs of percussion, meaning that the removal scars were not necessarily directed toward shaping the specific platform. Minute scars along the edge of the striking platform toward the debitage surface show additional treatment of the core face before the removal of blanks. In Layer R63 these minute scars are more common on the thick plain platforms (50.2%) than on faceted ones (16.4%) with a significant statistical difference (χ^2^ = 24.139, df = 1, p<0.0001). The striking platforms are mostly large (>3mm thick) (Table 3) with those of Bed J82BS both thicker and wider than those of Bed J83B1 (t = 5.687, df = 361, p<0.001; t = 4.587, df = 331, p<0.001, respectively) and Layer R63 (t = 9.016, df = 322, p<0.001; t = 8.082, df = 319, p<0.001, respectively). Statistically significant differences were also found between Beds J83B1 and Layer R63 (thickness: t = 4.578, df = 763, p<0.001; width: t = 4.902, df = 747, p<0.001, respectively).

**Table 3:** Frequencies of striking platform types (including blanks and tools).

		large plain	thin plain	multi scarred	dihedral	faceted	natural	N =	Thickness (mm)	s.d.	Width (mm)	s.d.
J82BS	PE flake	44.1	5.9	5.9	7.4	5.9	30.9	68	10.3	4.9	27.3	12.8
J82BS	flake	46.4	4.8	12.0	9.6	17.6	9.6	125	10.7	6.7	26.9	11.5
J82BS	NBK-flake	61.4	2.3	6.8	4.5	13.6	11.4	44	10.9	6	25.0	15.0
J82BS	*d*é*bordant*	31.3	6.3	12.5	25.0	18.8	6.3	16	10.1	5.0	23.8	12.1
J83B1	PE flake	53.5	2.3	13.2	3.9	12.4	14.7	129	8.1	4.5	21.1	10.0
J83B1	flake	42.6	9.0	16.0	5.9	19.5	7.0	256	7.6	4.9	22.0	10.4
J83B1	NBK-flake	47.6	2.4	11.9	2.4	21.4	14.3	42	8.1	3.9	22.5	18.6
J83B1	*d*é*bordant*	53.1	9.4	18.8	0.0	15.6	3.1	32	9.4	5.1	19.4	9.4
R63	PE flake	50.0	10.8	8.1	14.9	6.8	9.5	74	6.2	3.4	17.8	8.9
R63	flake	44.1	16.4	11.7	8.6	13.7	5.5	256	6.2	4.1	17.9	10.1
R63	NBK-flake	49.1	3.6	7.3	9.1	16.4	14.5	55	7.5	3.2	18.9	7.7
R63	*d*é*bordant*	33.3	8.3	16.7	16.7	20.8	4.2	24	8.2	4.8	19.5	10.1

The directions of dorsal removals (Table 4) on primary element flakes vary, with unidirectional scars originating at the platform end of the flake being the most common. Not surprisingly the plain flakes (with less cortex) show a more diverse distribution of scar patterns. Differences between the three layers were found in the case of flakes, with multi-directional scar patterns more common in Beds J82BS (χ^2^ = 7.642, df = 1, p = 0.006) than in Layer R63.

**Table 4:** Frequencies of scar patterns (including blanks and tools).

	item type	unidirectional	straight and perpendicular	bidirectional	lateral	opposed	multi directional	double ventral	n =
J82BS	PE flake	34.5	13.8	3.4	31.0	10.3	6.9		29
J82BS	Flake	35.4	16.5	2.5	3.8		41.8		79
J82BS	NBK-flake	46.4	25.0		10.7		17.9		28
J82BS	*débordant*		44.4		11.1		44.4		9
J83B1	PE flake	49.3	13.4		20.9	4.5	11.9		67
J83B1	Flake	34.5	25.4	3.5	1.4		33.8	1.4	142
J83B1	NBK-flake	63.3	13.3	3.3	10.0		10.0		30
J83B1	*débordant*	6.9	48.3		6.9		37.9		29
R63	PE flake	57.1	26.5	2.0	6.1	2.0	6.1		49
R63	Flake	32.2	28.7	9.1	2.1		23.1	4.9	143
R63	NBK-flake	47.1	23.5	5.9	11.8		11.8		34
R63	*débordant*	5.9	58.8		5.9		29.4		17

End terminations differ among the blanks and assemblages (Table 5). Of note is the relatively high percentage of items with hinge terminations: 19.8-21.9% for primary flakes and 24.5–35.3% for other flakes. Hinged terminations are more common among the plain flakes than any of the other items. In accounting the three assemblages the differences between plain flakes and PE flakes (χ^2^ = 4.670, df = 1, p = 0.031), flakes and NBK-flakes (χ^2^ = 5.212, df = 1, p = 0.022) as well as flakes and *débordants* (χ^2^ = 4.824, df = 1, p = 0.028) are statistically significant.

**Table 5:** Frequencies of end termination (including blanks and shaped items).

		feathered	hinged	overpassed	n =
82BS	PE flake	61.1	20.4	18.5	54
82BS	Flake	45.9	35.3	18.8	85
82BS	NBK-flake	42.4	15.2	42.4	33
82BS	*Débordant*	27.3	9.1	63.6	11
83B1	PE flake	55.2	19.8	25.0	96
83B1	Flake	62.5	24.5	13.0	216
83B1	NBK-flake	62.2	18.9	18.9	37
83B1	*Débordant*	56.7	20.0	23.3	30
R63	PE flake	65.6	21.9	12.5	64
R63	Flake	63.1	30.5	6.4	187
R63	NBK-flake	60.0	16.7	23.3	30
R63	*Débordant*	68.8	6.3	25.0	16

Metrics are presented in Table 6. Although Yabrudian assemblages are known for the large sizes of some tools, the blanks from all assemblages average only around 50 mm in length. Blanks from Bed J82BS are largest and those of Layer R63 are the smallest, with a statistically significant difference (Table 7).

**Table 6:** Metrics (blanks and shaped items).

		82BS		83B1	R63
		mm	s.d.	mm	s.d.	mm	s.d.
PE flake	L	56.9	11.7	52.1	15.6	46.3	13.8
	W	47.2	16.7	41.0	10.1	35.3	8.9
	T	16.5	5.8	14.1	4.9	12.9	5.3
	L/W	1.3	0.3	1.3	0.4	1.3	0.4
	W/T	3.1	1.1	3.3	1.1	3.3	1.2
flake	L	55.1	19.1	46.7	13.9	38.5	12.8
	W	48.6	20.2	38.6	10.0	31.5	9.0
	T	15.7	7.7	11.8	5.2	9.9	5.0
	L/W	1.2	0.4	1.2	0.4	1.3	0.4
	W/T	3.7	1.5	4.1	1.6	4.3	1.9
NBK-flake	L	63.1	20.0	54.9	12.9	50.2	15.5
	W	46.3	17.4	38.1	8.9	34.6	9.3
	T	16.8	5.9	13.7	5.4	13.2	5.1
	L/W	1.4	0.3	1.5	0.3	1.5	0.3
	W/T	2.8	0.9	3.2	1.0	3.0	1.1
*débordant*	L	55.8	9.7	55.0	15.0	45.0	9.6
	W	43.5	22.1	36.7	10.8	35.2	11.3
	T	18.0	5.7	15.4	5.2	12.8	4.2
	L/W	1.5	0.4	1.6	0.4	1.4	0.4
	W/T	2.5	1.0	2.5	0.7	3.0	0.9

L: Length; W: width; T: thickness.

**Table 7:** T-test of metrics (blanks and shaped items).

			t =	df =	p
PE flake	length	J82BS vs R63	4.305	105	<0.001
PE flake	width	J82BS vs R63	4.196	55	<0.001
PE flake	thickness	J82BS vs R63	4.274	170	<0.001
flake	length	J82BS vs R63	7.522	133	<0.001
flake	width	J82BS vs R63	7.433	96	<0.001
flake	thickness	J82BS vs R63	7.889	186	<0.001
NBK-flake	length	J82BS vs R63	2.734	58	0.008
NBK-flake	width	J82BS vs R63	3.139	49	0.003
NBK-flake	thickness	J82BS vs R63	3.244	92	0.002
*débordant*	length	J82BS vs R63	2.858	28	0.008
*débordant*	width	J82BS vs R63			
*débordant*	thickness	J82BS vs R63	3.265	37	0.002
PE flake	length	J82BS vs 83B1	2.127	119	0.035
PE flake	width	J82BS vs 83B1	2.218	54	0.031
PE flake	thickness	J82BS vs 83B1	3.237	208	0.001
flake	length	J82BS vs 83B1	3.816	134	<0.001
flake	width	J82BS vs 83B1	4.318	100	<0.001
flake	thickness	J82BS vs 83B1	5.236	192	<0.001
NBK-flake	length	J82BS vs 83B1	2.037	67	0.046
NBK-flake	width	J82BS vs 83B1	2.229	49	0.030
NBK-flake	thickness	J82BS vs 83B1	2.596	87	0.011
*débordant*	length	J82BS vs 83B1			
*débordant*	width	J82BS vs 83B1			
*débordant*	thickness	J82BS vs 83B1			
PE flake	length	J83B1 vs R63	2.382	163	0.018
PE flake	width	J83B1 vs R63	3.607	152	<0.001
PE flake	thickness	J83B1 vs R63			
flake	length	J83B1 vs R63	6.077	400	<0.001
flake	width	J83B1 vs R63	7.37	388	<0.001
flake	thickness	J83B1 vs R63	4.38	537	<0.001
NBK-flake	length	J83B1 vs R63			
NBK-flake	width	J83B1 vs R63			
NBK-flake	thickness	J83B1 vs R63			
*débordant*	length	J83B1 vs R63	2.391	45	0.021
*débordant*	width	J83B1 vs R63			
*débordant*	thickness	J83B1 vs R63	2.029	55	0.047

**Figure 6:**
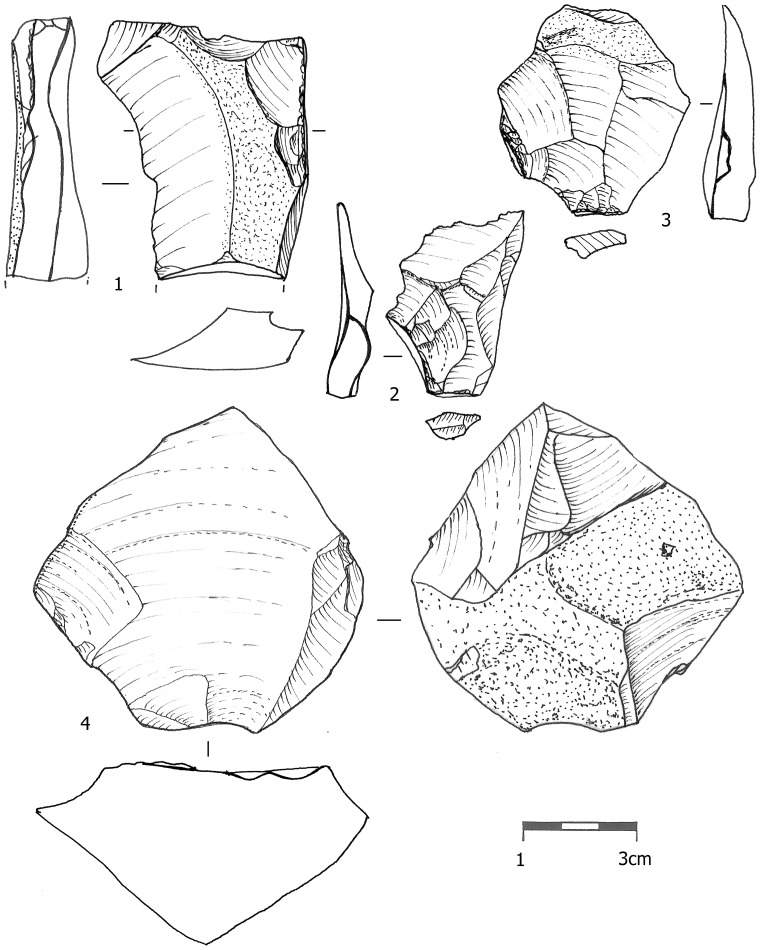
An initial *débordant* (1), a flat surface overpass items (2-3) and a centripetal core (4) from layer R63.

Core trimming elements (CTEs) from the three assemblages are presented in Table 8. The most distinct CTE type is the *débordant* (Fig. 5). We further identify two additional types which are very similar to *débordants* and resulted from similarly-shaped cores. One is the ‘flat surface overpass' (FSOP) which refers to flakes that reduced a segment of the plane of intersection of the core, bearing some remnant of a ridge (Fig. 6:2–3). In contrast to the typical *débordants*, these items are either flat, or removed only small segment of the lateral edge. A quarter of the FSOPs reduced a segment of the opposite edge of the core and not the lateral edge. The second type is the 'initial *débordant*' (IDEB), which resembles a *débordant* in having been removed from the lateral edge of a core, but which has a dorsal face covered with cortex indicating that it was one of the first removals of the debitage surface (Fig. 6:1). The *débordant*s' features including striking platforms, metrics and scar pattern are presented in Tables 3, 4, 5, 6, 7. The percentages of these three lateral CTE types is higher in Beds J82BS (61.4%) and J83B1 (69.6%) than in Layer R63 (48.1%) with a statistically significant difference between J83B1 and R63 (χ^2^ = 7.268, df = 1, p = 0.007). The lateral crest on the *débordants* is more often placed on the left side (Table 9).

**Table 8:** Core trimming elements.

		J82BS	J82BS	J82BS	J82BS	J82BS	J82BS	J83B1	J83B1	J83B1	J83B1	J83B1	J83B1	R63	R63	R63	R63	R63	R63
		whole	proximal	non-proximal	sum	%	%	whole	proximal	non-proximal	sum	%	%	whole	proximal	non-proximal	sum	%	
debordant	b	5	2		7	43.8		18	1		19	51.4		13	1	1	15	55.6	
	c-o-f					0.0		1			1	2.7		3			3	11.1	
	T	9			9	56.3		13	1	3	17	45.9		9			9	33.3	
	sum	14	2		16	100	28.1	32	2	3	37	100	40.2	25	1	1	27	100	34.2
overpass	b	3			3	37.5		1		2	3	33.3		3			3	37.5	
	c-o-f					0.0		1			1	11.1					0	0.0	
	T	4		1	5	62.5		4		1	5	55.6		4		1	5	62.5	
	sum	7		1	8	100	14.0	6	0	3	9	100	9.8	7	0	1	8	100	10.1
crested blade	b	1			1	50		2			2	100		1		1	2	100	
	c-o-f					0					0	0					0	0	
	T	1			1	50					0	0					0	0	
	sum	2			2	100	3.5	2	0	0	2	100	2.2	1	0	1	2	100	2.5
FSOP	b	7			7	70.0		10		1	11	50.0		7			7	87.5	
	c-o-f					0.0		1			1	4.5					0	0.0	
	T	3			3	30.0		9		1	10	45.5		1			1	12.5	
	sum	10			10	100	17.5	20	0	2	22	100	23.9	8	0	0	8	100	10.1
IDEB	b	1			1	11.1		2			2	40.0		1		1	2	66.7	
	c-o-f				0	0.0					0	0.0					0	0.0	
	T	8			8	88.9		1	1	1	3	60.0		1			1	33.3	
	sum	9	0	0	9	100	15.8	3	1	1	5	100	5.4	2	0	1	3	100	3.8
CTE-VARIA	b	4		1	5	41.7		7	1	2	10	58.8		19		4	23	74.2	
	c-o-f	1			1	8.3		3			3	17.6		1			1	3.2	
	T	6			6	50.0		4			4	23.5		6	1		7	22.6	
	sum	11	0	1	12	100	21.1	14	1	2	17	100	18.5	26	1	4	31	100	39.2
sum	b	21	2	1	24	42.1		40	2	5	47	51.1		44	1	7	52	65.8	
	c-o-f	1			1	1.8		6			6	6.5		4			4	5.1	
	T	31		1	32	56.1		31	2	6	39	42.4		21	1	1	23	29.1	
	sum	53	2	2	57	100	100	77	4	11	92	100	100	69	2	8	79	100	100

(FSOP: flat surface overpass item; IDEB: Initial debordant).

**Table 9:** Frequencies of side of blanks removed from the lateral edges of the cores (blanks and shaped).

	J82BS	J83B1	R63
	left	right	n =	left	right	n =	left	right	n =
*débordant*	60.0	40.0	15	64.7	35.3	34	69.6	30.4	23
NBK-flake	59.1	40.9	44	40.8	59.2	49	67.8	32.2	59
FSOP	44.4	55.6	9	50.0	50.0	14		40	5
IDEB	66.7	33.3	9	40.0	60.0	5	33		3
sum	58.4	41.6	77	50.0	50.0	102	66.7	33.3	90

Several types of cores were recognized in the three assemblages studied, differing in the number of striking platforms and their arrangements (Table 10). Core metrics are presented in Table 11. The single striking platform cores appear in various shapes: some bear blade scars or a combination of blade and flake scars (n = J82BS: 3; J83B1: 5; R63: 2), which is also typical of blade production in the Amudian of Tabun [17, pp. 147–226]. Most of the single striking platform cores however bear only flake scars. Only minor preliminary shaping is witnessed on these cores, and approximately half of them are fully covered by cortex aside from the debitage surface and striking platform.

**Table 10:** Core types.

	J82BS	J82BS	J83B1	J83B1	R63	R63
	n =	%	n =	%	n =	%
tested raw material	2	5.4	5	9.3	5	9.3
single striking platform	9	24.3	14	25.9	8	14.8
two striking platforms					2	3.7
Multi-platform	11	29.7	13	24.1	24	44.4
centripetal	15	40.5	21	38.9	15	27.8
unidirectional broad surface			1	1.9		0.0
sum	37	100	54	100	54	100
broken	16		14		10	
raw material			1		3	

(2 of the cores of J82BS were transformed into tools and are recorded within the core-tools in Table 1).

**Table 11:** Metrics of cores.

		length	s.d.	width	s.d.	thickness	s.d.
J82BS	single striking platform	56.4	22.4	53.7	26.7	40.4	16.0
J82BS	centripetal	60.4	14.4	55.5	14.7	28.3	7.6
J82BS	Multi-platform	62.7	15.4	47.8	15.9	35.7	12.4
J83B1	single striking platform	53.4	17.0	46.4	15.2	36.3	13.5
J83B1	centripetal	62.2	15.3	49.6	11.2	27.3	9.0
J83B1	Multi-platform	50.1	11.1	37.2	6.3	28.9	5.4
R63	single striking platform	47.9	12.0	46.5	11.3	26.9	5.9
R63	centripetal	52.2	13.1	46.8	11.9	21.4	9.2
R63	Multi-platform	57.2	13.5	42.7	12.2	27.1	8.8

Cores exhibiting exploitation typical of *débitage facial* (e.g. [68]–[69]), in which the product removals concentrate on the widest surface of the core, include broad surface unidirectional cores (n = 1) and cores with centripetal or partial centripetal removals (n = 51; Figs. 6:4; 7, 8). The centripetal cores are characterized by two surfaces, with the lower usually being angular and the upper surface flat or slightly convex. Cortex generally covers less than 20% of the core surface and is commonly confined to the under-surface. The number of scars preserved on the upper surface ranges from 3 to 16, with most cores characterized by multi-directional scar origins. Overpass scars are few, appearing on 16.7% of the centripetal cores. Scars of *débordant* or NBK removals (overpassing the lateral edge) on the other hand are very common, being observed on 42.9% of the centripetal cores. On 37.5% of the centripetal cores only one face was used for flake production, and in more than half (60.0%) of these particular cores the large flakes were removed parallel to the line of intersection between the two faces.

**Figure 7:**
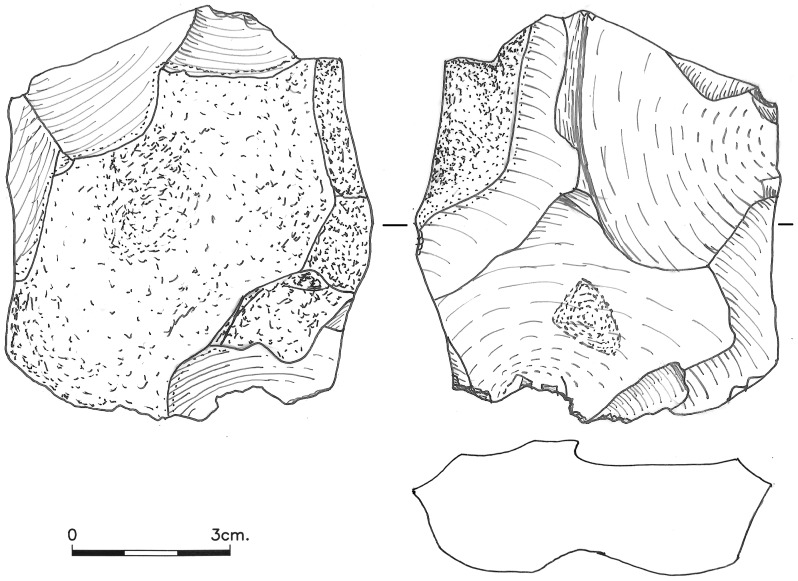
A centripetal core from Bed J83B1.

**Figure 8:**
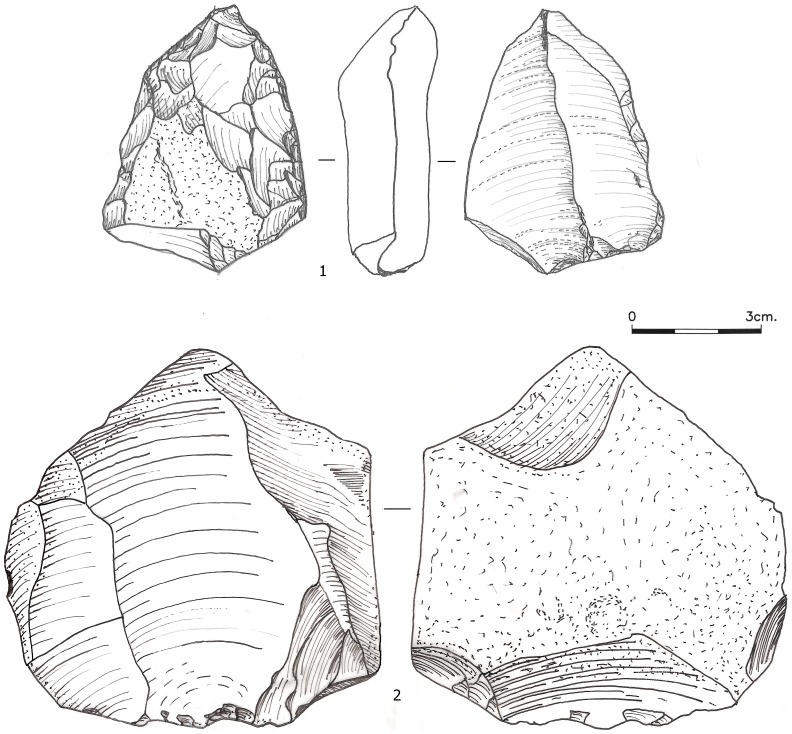
Broad surface unidirectional cores from Layer 75, core 1 was shaped into a scraper following discard (Although the items illustrated are not form the studied sample they demonstrate the discussed technological features).

As is typical for Yabrudian assemblages, a large percentage of the blanks from the three assemblages were shaped by retouch (Table 1). Retouched pieces are especially common within Beds J82BS (62.6%) and J83B1 (44.4%), but are significantly less frequent in Layer R63 (χ^2^ = 102.882, df = 1, p<0.0001; χ^2^ = 28.340, df = 1, p<0.0001). The difference in retouch frequency between Beds J82BS and J83B1 is also significant (χ^2^ = 32.747, df = 1, p<0.0001). Importantly, the highest percentages of secondary modification occur among the primary element flakes and NBK-flakes. CTEs were also commonly shaped into tools (Table 8).

**Figure 9:**
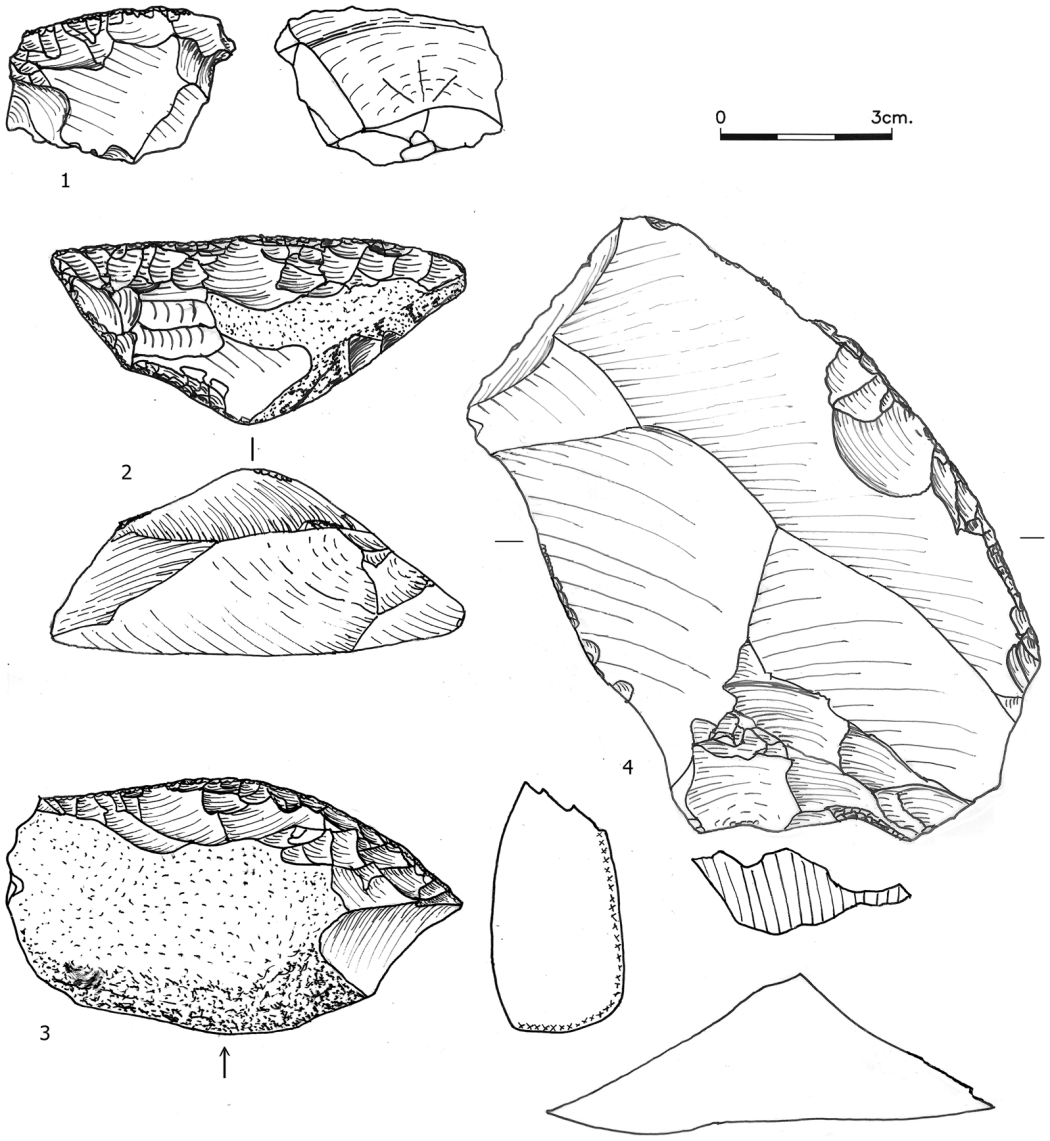
Scrapers from Bed J82BS and J83B1. No. 2 was shaped on a core.

**Figure 10:**
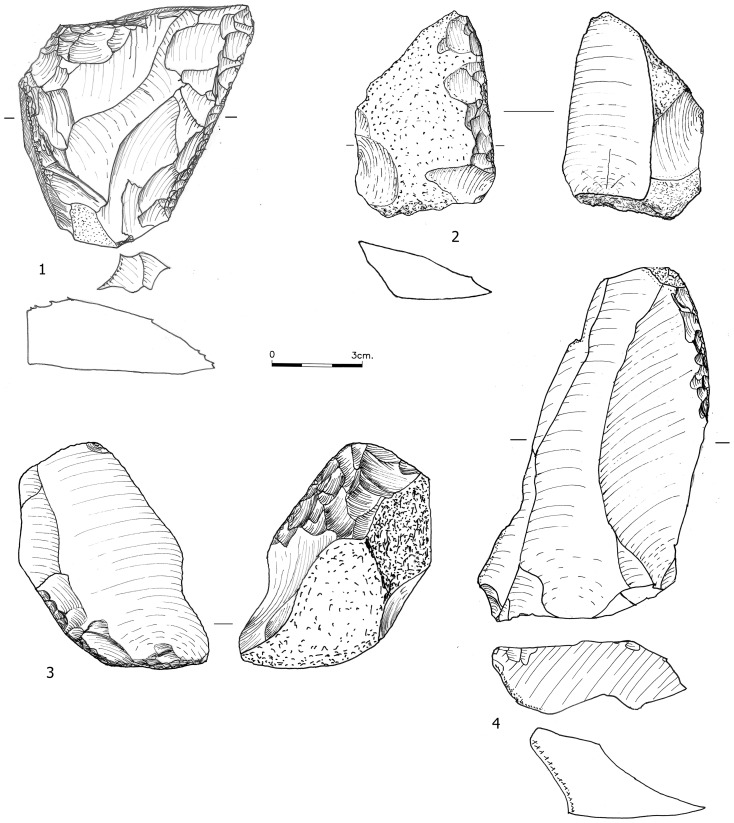
Scrapers from Bed J82BS shaped on *débordant* (1), initial *débordants* (2–3) and NBK-flake (4).

**Figure 11:**
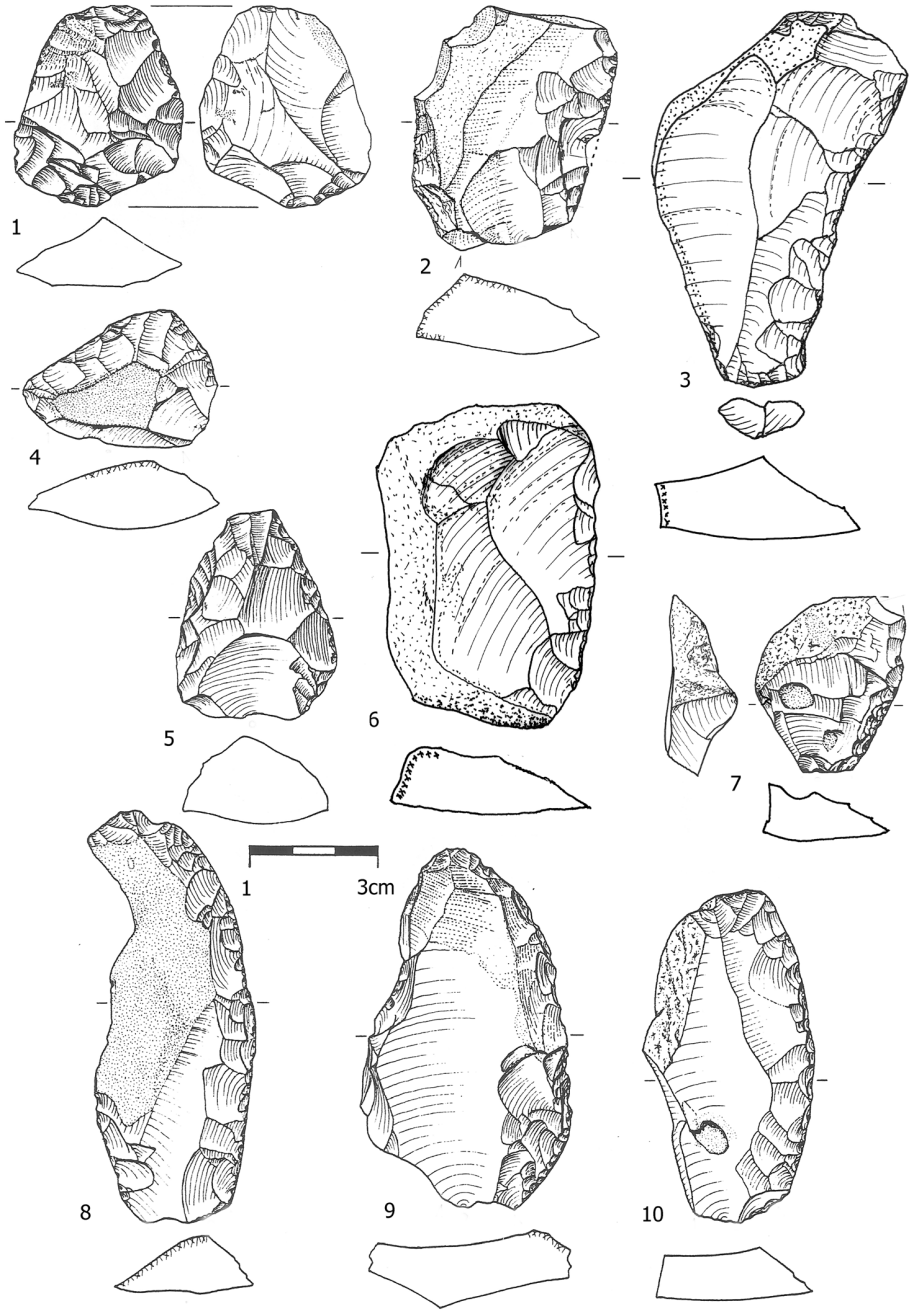
Scrapers from Layer R63. No. 1 was also utilized as a core on flake.

The retouched tool assemblages of the Yabrudian (Table 12) are dominated by scrapers (Figs. 9, 10, 11), many of which bear Quina retouch; in Bed J82BS almost all tools are scrapers (76.8%). The higher frequency of scrapers in Bed J82BS compared to J83B1 and R63 is statistically significant (χ^2^ = 19.157, df = 1, p<0.0001; χ^2^ = 55.923, df = 1, p<0.0001 respectively). No major differences in the composition of the scraper sub-types were identified among the assemblages (Table 13).

**Table 12:** Tools.

	J82BS	J82BS	J83B1	J83B1	R63	R63
	n =	%	n =	%	n =	%
single scraper	114	45.6	107	28.8	58	22.6
double scraper	18	7.2	17	4.6	19	7.4
convergent	8	3.2	12	3.2	1	0.4
dejete	20	8.0	29	7.8	15	5.8
bifacial scraper		0.0	6	1.6		0.0
ventral scraper	1	0.4	15	4.0	2	0.8
transversal	30	12.0	33	8.9	9	3.5
scraper fragment	1	0.4	1	0.3	8	3.1
retouched blade		0.0	8	2.2	3	1.2
retouched flake	31	12.4	74	19.9	41	16.0
notch denticulate	15	6.0	26	7.0	30	11.7
backed knife	1	0.4	2	0.5	3	1.2
end scraper		0.0	2	0.5	11	4.3
burin	2	0.8	7	1.9	31	12.1
chopper		0.0	0	0.0		0.0
handaxe	9	3.6	31	8.4	11	4.3
unidentified tool fragment		0.0	1	0.3	15	5.8
sum	250	100	371	100	257	100
all scrapers	192	76.8	220	59.3	112	43.6
"UP tools"	3	1.2	19	5.1	48	18.7

Two of the single scrapers of 82BS are made on cores (listed in Table 1 as core-tools).

**Table 13:** Division of scraper types.

	J82BS	J82BS	J83B1	J83B1	R63	R63
	n =	%	n =	%	n =	%
single scraper	114	59.7	107	48.9	58	55.8
double scraper	18	9.4	17	7.8	19	18.3
convergent	8	4.2	12	5.5	1	1.0
dejete	20	10.5	29	13.2	15	14.4
bifacial scraper	0	0.0	6	2.7	0	0.0
ventral scraper	1	0.5	15	6.8	2	1.9
transversal	30	15.7	33	15.1	9	8.7
sum	191	100	219	100	104	100

The higher presence of 'Upper Paleolithic tool types' in Layer R63 is also of note and it is statistically different from Beds J82BS and J83B1 (χ^2^ = 40.511, df = 1, p<0.0001; χ^2^ = 15.167, df = 1, p<0.0001 respectively). Among the 'Upper Paleolithic tool types' it is the burins that show the strongest difference between the assemblages. Many of the burins can be defined as Adlun burins which are typical of the Acheulo-Yabrudian complex [70]. In these artifacts the burin spall removal is obliquely oriented to the item's face (ventral or dorsal), forming a potential cutting edge ([17], pp: 67–69).

Handaxes are present in all assemblages, and are especially abundant in Bed J83B1. Seven of the handaxes (J82BS: 1; J83B1: 5; R63: 1) are represented by small fragments only. Since the handaxes are not part of the scraper-blank production they are not described here in detail. In general, their character matches former descriptions [51]–[53], [57]. Seven of the handaxes show evidence of recycling (J82BS: 1; J83B1: 4; R63: 2). Preferential flakes [71] were removed from five of them. Two bifacial pieces were also exploited as blade cores utilizing their narrow edges.

Another utilization of the blanks is represented by the 'cores on flakes' (Table 1), which are significantly more common in Layer R63 than in Bed J83B1 (χ^2^ = 7.865, df = 1, p = 0.005). Similar removals are also found on some of the items recorded as tools (J82BS: 5.5%; J83B1: 3.6%; R63: 11.4%; Fig. 11:1), either representing some additional complementary use or signifying the recycling of tools.

Various items detached from tools (burins, handaxes and scrapers), as well as from 'cores on flakes' (overpassed items, which removed either the distal or lateral edge of the flake they were removed from) were recorded as spalls. In layer R63 a procedure of rejuvenating the active edge of the scrapers by an elongated removal (burin spall like) was common.

### Yabrudian scraper-blank selection

Comparison of scrapers and unmodified blanks provides a general perspective on the characteristics of the blanks selected for further shaping into scrapers. In terms of different blank types secondarily modified, scrapers were mostly made on primary element flakes and plain flakes. However, plain flakes show the lowest percentage of retouch and NBK-flakes the highest (Table 14). This discrepancy reflects the very high proportion of plain flakes in the assemblage.

**Table 14:** Percentage of main blank types transformed into scrapers.

	J82BS	J83B1	R63
PE flake	57.0	37.2	20.2
flake	43.7	30.4	13.4
NBK-flake	68.1	39.2	27.4
*débordant*	50.0	35.1	22.2

Examination of the cortex type indicates that patinated surfaces were generally rejected for retouch and that there was a preference for blanks with calcareous cortex (Table 15). Primary element flakes with extensive cortical surface were more commonly selected for scrapers. For this comparison we compared blanks with a cortical cover of' ≥80% to the scrapers in which the entire dorsal face is covered by cortex aside from retouch. Furthermore, flakes with traces of cortex (≤20%) were more commonly selected for scrapers than flakes with no cortex at all. Evidence of hafting has not been found to date in Acheulo-Yabrudian assemblages [72]. Blank selection and shaping the tools most likely was a response to their suitability for manual prehension. Cortex, and especially calcareous cortex is of potential benefit in hand-held tools, much less slippery than patinated surfaces or flake scars.

**Table 15:** Comparison between blanks and scrapers.

		blank			scraper		n =	x^2^	df	p
PE flake										
**cortex type**		**calcareous**	**patinated**	**n = **	**calcareous**	**patinated**				
J82BS	n =	25	1	26	48	1	49			
J82BS	%	96.2	3.8	100	98.0	2.0	100			
J83B1	n =	40	13	53	43	3	46	4.639	1	0.031
J83B1	%	75.5	24.5	100	93.5	6.5	100			
R63	n =	50	14	64	23	3	26			
R63	%	78.1	21.9	100	88.5	11.5	100			
**full cortex cover**		**full**	**partial**	**n = **	**full**	**partial**				
J82BS	n =	5	19	24	28	21	49	7.171	1	0.007
J82BS	%	20.8	79.2	100	57.1	42.9	100			
J83B1	n =	17	46	63	35	22	57	13.07	1	<0.001
J83B1	%	27.0	73.0	100	61.4	38.6	100			
R63	n =	6	40	46	5	15	20			
R63	%	13.0	87.0	100	25.0	75.0	100			
Flake										
**cortex (<30% of dorsal face)**		**no cortex**	**cortex**	**n = **	**no cortex**	**cortex**				
J82BS	n =	35	20	55	30	39	69	4.211	1	0.040
J82BS	%	63.6	36.4	100	43.5	56.5	100			
J83B1	n =	134	40	174	55	33	88	5.422	1	0.020
J83B1	%	77.0	23.0	100	62.5	37.5	100			
R63	n =	183	54	237	33	20	53	4.338	1	0.037
R63	%	77.2	22.8	100	62.3	37.7	100			

Feathered end terminations are often considered indicative of “successful” flake production. However, in the three assemblages flakes with hinged or overpassed terminations were favored slightly for conversion into scrapers and feather terminations are more abundant among unretouched pieces (J82BS: blanks: 54.3%, tools: 40.8%; J83B1: blanks: 66.4%, tools: 56.6%; R63: blanks: 65.1%, tools: 55.3%). In uniting the three assemblages this difference is statistically significant (χ^2^ = 6.984, df = 1, p = 0.008). This also might be related to prehension, in that both overpassed and hinged terminations are blunt and more easily and safely gripped in the hand [e.g. 73].

As is typical in Paleolithic assemblages, larger blanks were selected for the shaping of scrapers (Table 16). While many of the scrapers show a convex profile of the ventral face, no clear difference was observed from the comparison between blanks and tools in this respect. It is most likely that this convex profile resulted from extensive reduction leaving most of the scraper’s remaining mass in the area of the bulb of percussion.

**Table 16:** Comparison between blanks and scrapers.

		blank	s.d.	scraper blank	s.d.	scraper	s.d.	compared*	*t*	*df*	*p*
**PE flake**											
J82BS	length	50.7	11.5	62.0	10.7	55.9	13.1	1 vs 2	3.334	41	0.002
J82BS	width	41.8	15.0	50.6	15.7	49.8	15.3				
J82BS	thickness	15.2	5.8	16.1	4.7	16.1	4.7				
J83B1	length	47.4	14.1	55.9	15.3	54.2	14.7	1 vs 2	2.633	85	0.010
J83B1	width	38.8	9.2	47.0	10.9	43.9	12.1	1 vs 2	2.941	69	0.004
J83B1	thickness	12.8	4.8	14.4	4.5	14.4	4.5				
R63	length	43.9	13.8	51.8	14.2	51.4	13.7	1 vs 3	2.052	62	0.044
R63	width	33.8	8.6	41.0	6.6	37.7	7.8				
R63	thickness	12.0	5.9	14.2	4.3	14.2	4.3				
**flake**											
J82BS	length	46.5	16.1	62.4	14.5	60.2	17.5	1 vs 2	4.271	71	<0.001
J82BS	width	41.9	15.4	52.7	12.7	48.6	14.4	1 vs 2	2.670	59	0.010
J82BS	thickness	12.1	5.6	16.9	6.6	16.9	6.6	1 vs 2	3.926	98	<0.001
J83B1	length	42.2	11.1	57.4	16.6	53.0	17.2	1 vs 2	5.925	64	<0.001
J83B1	width	37.1	9.2	43.4	12.2	42.4	12.3	1 vs 2	3.084	160	0.002
J83B1	thickness	9.7	4.1	14.3	5.2	14.3	5.2	1 vs 2	7.322	161	<0.001
R63	length	36.8	12.9	47.0	8.6	50.0	13.1	1 vs 2	2.899	159	0.004
R63	width	30.5	9.1	36.3	8.5	38.8	8.0	1 vs 3	5.386	190	<0.001
R63	thickness	7.9	3.8	13.0	4.6	13.0	4.6	1 vs 2	7.604	199	<0.001

Scraper blank: Referring to cases were original measures were left and not for cases were abrupt retouch is present.

*1: blank; 2: scraper-blank; 3: scraper.

As an aggregate of characteristics, elements which affect the manual prehension of the items were central to selection of blanks for modification. This is exemplified by size and end terminations, and more significantly in the items that show a higher percentage of selection for scraper shaping: PE flakes, NBK-flakes and *débordants*. Moreover, retouch was usually placed so that the modified edge was opposite a thick and usually cortical margin.

### Yabrudian scraper-blank technology of Tabun

Using the characteristics of the blanks, CTEs, cores and tools we suggest the following general scheme for the production of Yabrudian scraper blanks. The selection of prime nodules with calcareous cortex was the first step of this reduction sequence (Table 2). Knapping was performed by hard hammer as indicated by the thick striking platforms (Table 3). Given the high percentage of scrapers made on cortical and lateral items (Tables 1, 15) it is apparent that Yabrudian scraper-blank technology focused not only on exploitation of the internal mass of the flint nodule but also on efficient exploitation of its outer surface. This tendency is also illustrated by the relatively low ratio between plain flakes and flakes with >30% dorsal cortex (PE flakes) (J82BS: 1.7; J83B1: 2.2; R63: 3.1; see also Table 15).

The first removal of large cortical items from the cores served both to create scraper blanks and to shape the core for further production. Generally, the initial removals were taken from the broadest face of the nodule, following the concept of *debitage facial*. We assume at least two fully cortical blanks were removed at this stage, one forming the main striking platform and one initiating the debitage surface. The fact that a natural striking platform appears on a substantial portion of the fully cortical PE flakes (J82BS: 23.1%; J83B1: 21.6%; R63: 12.5%), typical for ‘initial flakes' [32, p. 141] supports the notion that several fully cortical flakes were often made from a single core. The 'initial *débordants'* represent the removal of lateral items from a partially cortical face, indicating that even at this stage, removals, either for production or shaping, were struck from more than one direction. This is further reflected in the flakes' scar patterns: the substantial percentage of PE flakes with laterally originating dorsal scars shows that the striking points for successive detachments often moved around the perimeter of the broad face of the core. Nevertheless, the fact that the PE flakes show fewer striking platforms composed of several scars (faceting, dihedral and multi-scarred) than the plain flakes (Table 3), with a statistical significance in the case of Beds J82BS (χ^2^ = 72519, df = 1, p = 0.007) and J83B1 (χ^2^ = 4.733, df = 1, p = 0.030), indicates that organized circumferential or partial circumferential striking platform were not constructed at this early stage.

Since we are not dealing with giant cores as in the Acheulean [33], the production of relatively large blanks required exploiting a large part of the core's surface area. In order to maintain production of such large blanks, the debitage surface must be relatively flat. Furthermore, it is difficult to remove relatively large flakes that exploit most of the core area from both core faces, so it was often necessary to maintain a hierarchy of surfaces, with the upper surface used for production and the lower surface used as a striking platform (e.g. [56], [74]). The first series of removals was organized such that the resulting core had two surfaces with a plane of intersection between them (e.g. [25]). Since the first removals focused on the production of large cortical flakes and not on "decortication" it is assumed that the nodule mass was substantially reduced already after the first series of removals.

The next phase of reduction includes the removal of blanks from both the center and lateral edges of the core face. The focus on large blanks means that low convexities of the flaking surface must be maintained through this phase of exploitation. The paucity of triangular flakes typical of discoidal or centripetal Levallois cores in the assemblage supports our reconstruction of blanks that exploit a large segment of the debitage surface (e.g. [25], [74]). In order to achieve this, blanks were removed essentially parallel to the plane of intersection between the core's two faces (e.g. [25], [56]).

The ratio of PE flakes and plain flakes to lateral items (NBKs, *débordants*, FSOP, ‘initial *débordants*') is 3.1 in Bed J82BS, 4.6 at Bed J83B1 and 5.3 at Layer R63 (these ratios are probably inflated because flakes and cortical flakes could be produced from almost any of the identified production schemes). The mean widths of the scrapers shaped on PE flakes, NBKs-flakes and *débordants* are similar to that of the discarded cores (compare Tables 11, 16). While it is clear that the sizes of the cores dramatically reduced, it still suggests that the detachment of 2–3 items often removed all or part of the debitage surface from side to side.

The removal of large blanks was primarily performed by striking far interior from the core face. The correlation between striking platform size and blank size [75]–[76] was clearly recognized by past knappers. Another advantage of this procedure was in manufacturing items that are thick throughout most of their profile (medial profile symmetry; [77, p.335]). It was common practice in Amudian assemblages to intentionally overpass blanks, which results in large products that possess thick end terminations [17], [38]. However, this strategy was not used often in the manufacture of scraper blanks, probably because overpassing also leads to a concave ventral surface, a shape which seems to not have been favored: as noted above, many of the scrapers have a convex ventral curvature. It is of note that the broad surface unidirectional cores that are found in small numbers in many of the Yabrudian assemblages of Tabun exhibit overpassing scars (Fig. 8:2). The removal of such overpassed items, which could have occurred at any step of the reduction, would have changed the core from centripetal to unidirectional. The paucity of these cores, as well as the small numbers of FSOPs which removed the opposite edge, suggests that it was either a seldom-used strategy, or that overpassing often led to abandonment or reshaping of cores.

A significant number of blanks are characterized by a hinged end-termination (Table 5). This is assumed to be an advantage for hand-held tools. The fact that it is significantly more common among the plain flakes than on any of the lateral items or the PE flakes suggests that it could have been a calculated procedure, perhaps because it provided an alternative gripping surface where cortex or other abrupt lateral edges were lacking. The continual removal of items with hinge terminations could only be accomplished by rotating the location of the striking platform. In this way, hinges from previous removals could be cleaned away rather than accumulating and inhibiting further production. The tendency for a left orientation for all lateral items and especially for *débordants* (Table 9) suggests that the rotation was more often conducted clockwise than in the opposite direction.

Since cortical blanks were often used in the Yabrudian, flipping sides of the cores for further production was a practical choice. The two core faces were worked probably sequentially rather than simultaneously. The centripetal cores with angular surfaces should be regarded as representing pieces abandoned following the loss of the necessary flat surface, or as the result of its exploitation for the extraction of small flakes. A transformation from a well-controlled core (e.g. Levallois) to a form showing a lesser degree of control (e.g. discoidal) as a final stage of production has been noted in several Middle Paleolithic industries (e.g. [56]). Since Yabrudian scraper-blank production was aimed at relatively large blanks, a core that had lost its potential to yield additional large, flat flakes might still contain a significant mass of flint. Thus, transformation of centripetal cores for simple small flake production is one way to extend their utility. We hypothesize that this sort of transformation often resulted in multi-platform cores. The notion that multi-platform cores are more highly reduced is supported by the fact that they are significantly less wide than centripetal cores (t = 3.216, df = 96, p = 0.002). The numerous 'cores on flakes' and the small multi-striking platform cores seem to have been intentionally exploited for making small flakes. This very economical exploitation of cores parallels the sometimes-extensive reduction of scrapers.

## Discussion and Conclusion

The Acheulo-Yabrudian complex demonstrates a high degree of variation within its three facies. Thus far predetermined debitage technology has been recognized for Amudian blade production [17], [38]. However, Amudian assemblages occur infrequently in sites of the Acheulo-Yabrudian complex [6]–[8], [15]. The exception is Qesem cave where Amudian assemblages are dominant [16]. While this blade production was practiced in the Yabrudian and Acheulean facies as well [17], it usually constitutes just a small part of these assemblages. The results of the current study of the three Yabrudian assemblages from Tabun show that the more common Yabrudian facies (e.g. [6]–[8], [13], [15]) and associated production of scraper-blanks is characterized by a very different system of reduction that still manifests a degree of planning and predetermination.

Although artifacts in the three presented assemblages are the outcome of several reduction sequences aimed at different products, the manufacture of scraper-blanks constitutes the bulk of the material: scrapers are the dominant tool type and typical scraper blanks are abundant in the assemblages. Some resharpenening of handaxes and recycling for use as cores also occurred at the site (e.g. [51]–[53]), but this did not significantly affect the general content of the assemblages. Blade production is also a minor component, represented by a few blades, crested blades, overpassed items and cores. Simple flake production, aimed at relatively small blanks, is the other main element in these assemblages, primarily represented by the single striking platform and multi striking platform cores. Some of these cores probably began life as larger centripetal cores for making scraper blanks but were repurposed in order to extend their use lives.

The typical Yabrudian scraper-blank reduction sequence in these assemblages of Tabun exhibits the following characteristics:

1. Selection of nodules with calcareous cortex: cortex was an essential part of the products.
2. Knapping was conducted with hard hammer and by striking well back from the edge of the striking platform.
3. Flakes were removed primarily from the widest surface of the raw material (*débitage facial*), forming an upper production surface and a lower striking platform with a plane of intersection between them.
4. Blank removal was parallel to the plane of intersection and the removal of lateral blanks alongside central blanks maintained the low convexities of the debitage surface.
5. The removal of flakes shifted along the core's circumference according to the character of the debitage surface. Instead of removing small flakes to adjust the debitage surface convexities, the knappers located another point at the core from which one or more large blanks could be removed. In this manner almost all items produced from the debitage surface are both predetermin*ed* and predetermin*ing*.
6. Many of the blanks produced, and most of the ones selected for retouch, had characteristics that enhanced their potential as hand-held tools: in particular, large size, thick edges, and cortical surfaces (e.g. NBK-flakes) that ensure a firm grip of the tools seem to have been preferred.
7. As a result of removing relatively large flakes the core mass diminished rapidly.
8. Cores of a relatively small size (ca. <5 cm) were often transformed into multi-striking platform cores from which less regular flakes were produced. Some discarded cores also became ‘blanks' for tools.
9. Because the reduction sequence exploits both the exterior and the interior mass of the cores, with few predetermin*ing* removals for preparing or maintaining the cores, the percentage of usable blanks and retouched pieces is high.

Although the three assemblages show many similarities, several patterns of change in the Yabrudian scraper-blank technology are recognized during the time frame investigated here, ca. 415–320 ky. There are only minor fluctuations among scraper sub-types. An increase in the use of pristine calcareous nodules is noted. In terms of blanks, there is an increase over time in the presence of cortical items, represented both in the percentages of PE flakes (Table 1) as well as in the percentages of flakes with partial cortical cover (Table 15). Blanks also show an increase in size (Table 7), which parallels an increase in striking platform size (Table 3). In terms of CTEs, lateral CTEs, including *débordants*, FSOPs and IDEBs, are more frequent in Beds J82BS and J83B1 than in Layer R63. Here however the trend is not linear and in fact Bed J83B1 shows a higher percentage than Bed J82BS. In terms of cores, centripetal forms become more frequent in time (Table 10). The higher percentage of flakes with multi-directional scar patterns is further evidence of this trend (Table 4).

Scrapers, and particularly Quina scrapers, have been reported from all Acheulo-Yabrudian sites, and in Yabrudian assemblages they usually constitute at least half of the tools (e.g. [8], [15]–[16], [41], [70]). Nevertheless, data for a comprehensive comparison of manufacture processes are scarce. Nonetheless, our findings from Tabun do not appear to be unique. The frequent use of cortical blanks has been mentioned for several of the sites (e.g. [41], [43], [50]). Thick platforms have been also long recognized as typical, however in contrast to former reports that emphasized plain striking platforms [8], [78], in Tabun we recognize also more elaborated types including faceted, multi scarred and dihedral striking platforms.

The ventral profile of the scrapers commonly tends to be convex as noticed for example by Shmookler [79, p. 14] in his study of the Yabrudian of Masloukh Layer C. Scrapers on cores were found in Yabrud I [8, p. 32], Adlun [70] and Dederiyeh [41, fig. 6:9] and in our experience many of the seeming bifacial scrapers (e.g. [80]) are scrapers made on cores or scrapers re-worked as cores on flakes. With reference to cores, of note is the presence of discodial cores or broad-surface unidirectional cores in many of the Yabrudian assemblages such as in Yabrud I [8, p.38, 83], Qesem [50], Adlun [15], Maslukh [78] and Hummal [81]. Although a comparative analysis of Yabrudian scraper-blank technology is still needed, the above comparisons support the existence of a common reduction sequence in Yabrudian assemblages.

Predetermination is usually ascribed not only as a marker of the high cognitive capabilities of the relatively more recent hominins (e.g. [31]) but also as a proxy for a more calculated strategy of resource exploitation, particularly with reference to mobility patterns (e.g. [77], [82]). Yabrudian scraper-blank technology does not show the same complexity and planning depth as the Levallois method [e.g. 25]. It does however show many important elements that bond it with the Levallois. In fact, it exhibits several of the criteria for identifying Levallois described by Boëda [25], albeit executed with less precision and delicacy. These include (1) two surfaces with a plane of intersection between them, (2) a hierarchy of the surfaces in which one serves as a striking platform while the other as a production surface, (3) production parallel to the plane of intersection, (4) removals perpendicular to the axis of the striking platform, and (5) reduction by hard hammer. Of the various characteristics of the Levallois extensive platform faceting and lateral and distal preparation of the flaking surface convexities are absent or expressed infrequently. In case of the latter, the control over the flaking surface convexities was performed by removing large blanks parallel the plane of intersection in a manner that resembles the concept of the recurrent Levallois (e.g. [25]).

It has been argued that the difference between discoidal cores and centripetal Levallois cores is a matter of degree, and that the two show the same general technological concept, differing only in the fine details of production and maintenance (e.g. [25], [83]–[84]). In the case of the Yabrudian of Tabun we are dealing with centripetal production from broad surface cores that bears many similarities with recurrent Levallois; here too, the differences seem to be a matter of degree rather than kind, as discussed also for the “proto-Levallois” by White and Ashton [85]. Whether the changes in Yabrudian scraper-blank technology over time moved the reduction system in the direction of Levallois requires further study of the Yabrudian at Tabun and other sites. It is already clear, however, that Yabrudian technology was not static. This may hint that a faster pace of change is evident within the Acheulo-Yabrudian than in the Acheulian culture of the Lower Paleolithic [86]–[87].

**Figure 12:**
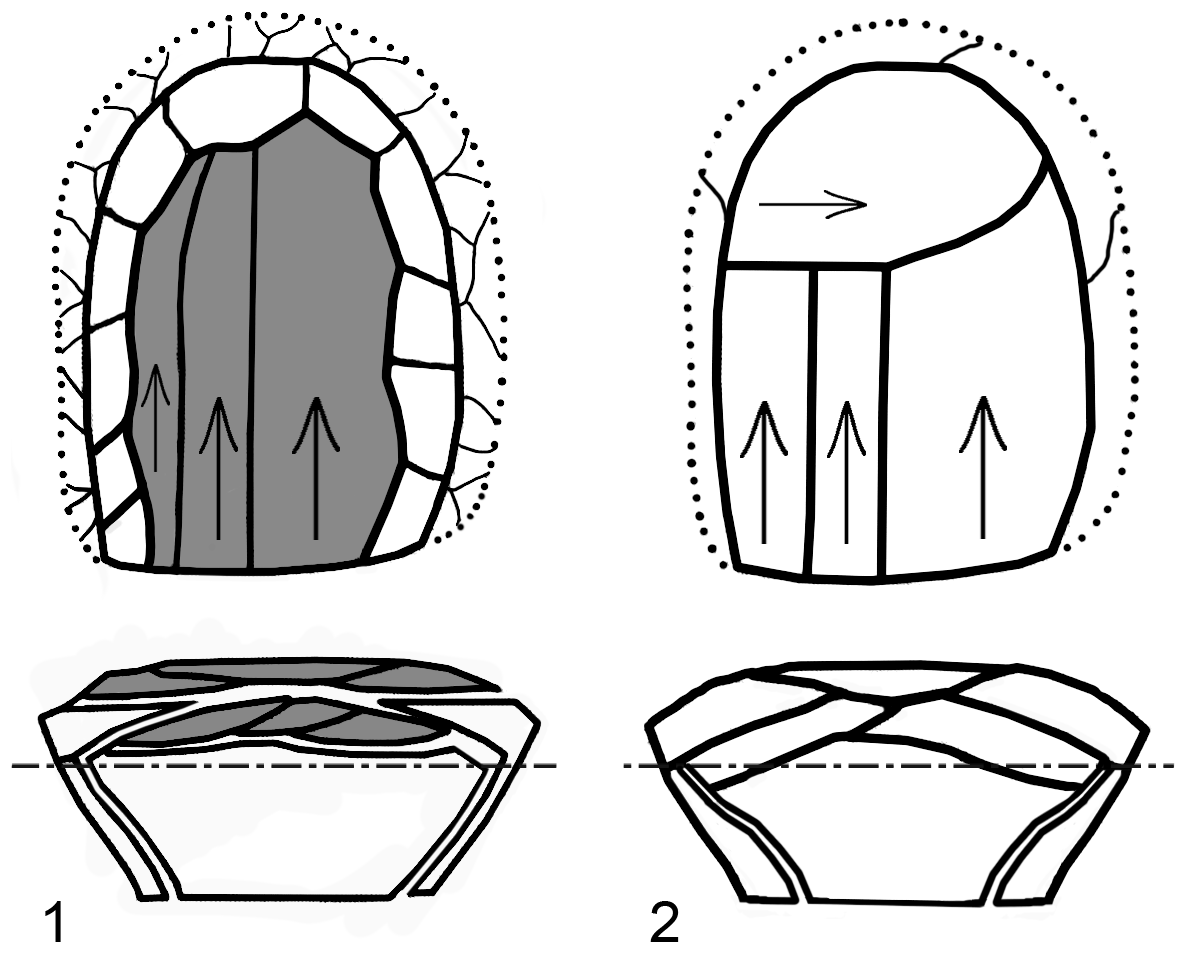
A schematic representation of the difference perception of reduction between Levallois (1) and the Yabrudian scraper-blank technology (2). (1) After Boëda et al. [26].

No one would deny that there are major differences between true Levallois and the Yabrudian scraper-blank technology, whether in the plane of intersection between the two surfaces, in the preparation of platforms, or in the efforts to shape surface convexities in order to achieve a precise blank shape. However there is also a major difference between the Yabrudian and the Levantine Mousterian industries in the extent of retouch on the blanks produced. While the Yabrudian is characterized by heavy retouch and reduction, the Levantine Mousterian is usually characterized by low frequency and light development of retouch (e.g. [77], [88]). Meignen et al. [82] marked this same difference among the industries of the Middle Paleolithic of Western Europe, arguing that the Quina and Levallois technologies are both predetermined to a certain degree. In the Quina industry, which resembles the Yabrudian in many respects, there is less emphasis on pre-shaping blanks and more use of supplementary retouch to achieve tool shape, whereas in Levallois industries the effort is on the production of blanks that do not need further shaping. This same distinction fits the Yabrudian and the early Levantine Mousterian which follows it. These two systems of production employed a recurrent reduction that encompassed both the center of the debitage surface as well as its edges in order to maintain the preferential convexities and the production in general (Fig. 12). However, with the Levallois method the internal part of the upper surface was the main locus of production and lateral removals of *débordants* or other items are considered as maintenance steps [25], or as a secondary source of usable blanks [88]. In contrast, with Yabrudian blank production the lateral items with cortical backs and surfaces seem to have been more important as tool blanks. The effects of the different procedures on core geometry and resulting blanks were surely recognized by the knappers of both periods, even if they made different choices about which elements of production to exploit.

Following this, an important difference between Levallois of the Middle Paleolithic and Yabrudian scraper-blank production simply relates to characteristics of the target products. The major differences between the Yabrudian and the Levallois Mousterian industries may reflect the use of hafting. Evidence of hafting in the Middle Paleolithic is abundant and increasing (e.g. [89]–[92]). Many studies attempted to calculate the differences in efficiency of flint mass exploitation between various industries throughout Paleolithic sequences (e.g. [77], [93]). Shea [77, p. 302] recently argued that "hafting is a *quantum leap* (our emphasis) in the time and energy allocated in preparation for tool use". The importance of this transformation was also recently highlighted by Barham [94]. We concur with these observations and further argue that changes in strategies of predetermination between Yabrudian and Levallois are a response to a shift in paradigm of how stone tools were used. A major constraint on blank production of the Yabrudian relates to the suitability of products for being held in the hand. In the Middle Paleolithic Mousterian, standardized size for hafting, especially as regards thickness of blanks, seems to be a more important constraint (e.g. [95]). The significance of blank size and cortex for improving handgrip was noted for Amudian blade production (e.g. [17], [38]). In fact, all industries of the Acheulo-Yabrudian complex took advantage of cortical surfaces in the shaping of tools. This is obvious for Yabrudian scrapers, but it is also apparent in the handaxes, many of which retain cortex, especially along their base [51]–[53]; [57], [96]–[97], a point discussed with more details by Gowlett [98]. In industries where hafting is still uncommon or totally lacking, the production of robust blanks versus delicate blanks should not be considered evidence for lack of ability or waste of raw material, but rather a response to ergonomic and functional considerations.

Of course, there are many ways to make hand-held tools. Yabrudian scraper-blank production is clearly oriented toward making heavy-duty tools that can and often were subjected to extensive reduction. Amudian blades, while also held in the hand, were not intended for intensive retouch and prolonged use. The same can be said of the small flakes that came from cores on flakes in Yabrudian assemblages. These systems embody another axis of variation in Paleolithic technologies; that of producing fresh edges by retouch as opposed to making more blanks. Whether the choice is arbitrary, related to artifact function, or stems from mobility and logistical decisions (e.g., transported tools) remains to be seen. The fact that there is so much variation within the Tabun sequence indicates that the different strategies were not strictly a response to lithic raw material availability. However, it does provide further evidence of the tactical and technical flexibility of Middle Pleistocene hominins.

The issues of cultural continuity between the Acheulo-Yabrudian and the Levallois-Mousterian in the Levant, and of the biological relations between the populations that made them, remain contentious. Opinions range from a clear break between the two indicating a possible introduction of a new population to the region (e.g. [4], [48]) to arguments for continuity, mostly based on the presence of blade production on either side of the boundary (e.g. [13], [99]–[100]). The results presented here indicate however that a comparison based on the presence or absence of particular forms of technology or products might be misleading. Any comparison of these two groups of assemblages must account for the possibility that the difference between the industries is complicated by a shift in how hominins exploited lithic raw material for tool use—a shift from exclusive use of hand-held tools, which seem to reach their peak in the Acheulo-Yabrudian in the Levant, to more frequent hafting in the Middle Paleolithic.
